# Establishment and validation of a ubiquitination-related gene signature associated with prognosis in pancreatic duct adenocarcinoma

**DOI:** 10.3389/fimmu.2023.1171811

**Published:** 2023-06-09

**Authors:** Yangyang Guo, Zhixuan Wu, Kenan Cen, Yongheng Bai, Ying Dai, Yifeng Mai, Kai Hong, Liangchen Qu

**Affiliations:** ^1^ Department of General Surgery, The First Affiliated Hospital of Ningbo University, Ningbo, China; ^2^ Key Laboratory of Diagnosis and Treatment of Severe Hepato-Pancreatic Diseases of Zhejiang Province, The First Affiliated Hospital of Wenzhou Medical University, Wenzhou, China; ^3^ National Key Clinical Specialty (General Surgery), The First Affiliated Hospital of Wenzhou Medical University, Wenzhou, China; ^4^ Department of Emergency, Taizhou Hospital of Zhejiang Province Affiliated to Wenzhou Medical University, Taizhou, Zhejiang, China

**Keywords:** ubiquitination, PDAC, immune microenvironment, immunotherapy, prognosis

## Abstract

**Background:**

Patients with pancreatic duct adenocarcinoma (PDAC) have varied prognoses that depend on numerous variables. However, additional research is required to uncover the latent impact of ubiquitination-related genes (URGs) on determining PDAC patients’ prognoses.

**Methods:**

The URGs clusters were discovered via consensus clustering, and the prognostic differentially expressed genes (DEGs) across clusters were utilized to develop a signature using a least absolute shrinkage and selection operator (LASSO) regression analysis of data from TCGA-PAAD. Verification analyses were conducted across TCGA-PAAD, GSE57495 and ICGC-PACA-AU to show the robustness of the signature. RT-qPCR was used to verify the expression of risk genes. Lastly, we formulated a nomogram to improve the clinical efficacy of our predictive tool.

**Results:**

The URGs signature, comprised of three genes, was developed and was shown to be highly correlated with the prognoses of PAAD patients. The nomogram was established by combining the URGs signature with clinicopathological characteristics. We discovered that the URGs signature was remarkably superior than other individual predictors (age, grade, T stage, et al). Also, the immune microenvironment analysis indicated that ESTIMATEscore, ImmuneScores, and StromalScores were elevated in the low-risk group. The immune cells that infiltrated the tissues were different between the two groups, as did the expression of immune-related genes.

**Conclusion:**

The URGs signature could act as the biomarker of prognosis and selecting appropriate therapeutic drugs for PDAC patients.

## Introduction

Pancreatic duct adenocarcinoma (PDAC)is often called the “king of cancer” (1, 2). Its prognosis is extremely poor making it the fourth leading contributor to cancer-associated death globally (3). Due to the lack of specific clinical manifestations in the first stages of PDAC, only a small proportion of individuals are identified with certainty at such early stages, and the vast majority are diagnosed at a more advanced level (4). Despite recent breakthroughs in the systematic treatment of PDAC, the prognoses for those with the advanced disease remain dismal owing to the disease’s rapid local progression and frequent distant metastasis (5). Therefore, it is very important to find some pivotal genes that may regulate the onset and advancement of PDAC and serve as novel therapeutic targets for PDAC.

Recently, with the considerable progress of cutting-edge high-throughput sequencing technologies and the growing improvement of public databases, two authoritative databases, the Cancer Genome Atlas (TCGA) and the International Cancer Genome Consortium (ICGC), have collected considerable clinical, pathological, and biological data of cancer patients (6, 7). Researchers can use these data and a variety of bioinformatics analysis methods to screen and predict new diagnostic and prognostic markers for various cancers. Like most cancers, PDAC is a complex malignant disease involving multiple molecules. At present, researchers have successfully established a variety of effective polygene prognostic risk models using bioinformatics technology (8, 9). A multigene prognosis model is helpful to evaluate the total survival period and recurrence risk of patients, and identify high-risk patients with poor prognosis and timely and systematic treatment, while for low-risk patients, unnecessary treatment burden can be appropriately avoided. For example, based on DNA methylation, autophagy, and immune-related genes, the prognosis prediction models for PDAC have strong prediction ability, which can be used for early diagnosis, prognosis evaluation, and treatment (10–12). Ubiquitination-related genes (URGs) have been reported as regulators of tumors, affecting tumor cell cycle regulation, gene expression, and progression (13). However, there is much less understanding of ubiquitination in the PDAC microenvironment and prognosis.

In this study, to examine the link between URGs and the prognosis of PDAC individuals, we used multivariate Cox and LASSO regression analyses to identify three ubiquitination genes that have the most impact on the prognosis of PDAC individuals and constructed a three genes prognosis model. In addition, we integrated clinicopathological parameters and risk scores to develop a novel nomogram for clinical application, which can more directly assess PDAC patients’ prognoses, and help achieve personalized therapy. We then found the different immune statuses between various URGs risk groups. Additionally, we examined the prognostic model’s biological roles and signaling pathways to further evaluate the probable molecular processes that influence PDAC patients’ survival and prognoses.

## Materials and methods

### Data retrieval

**Figure 1:**
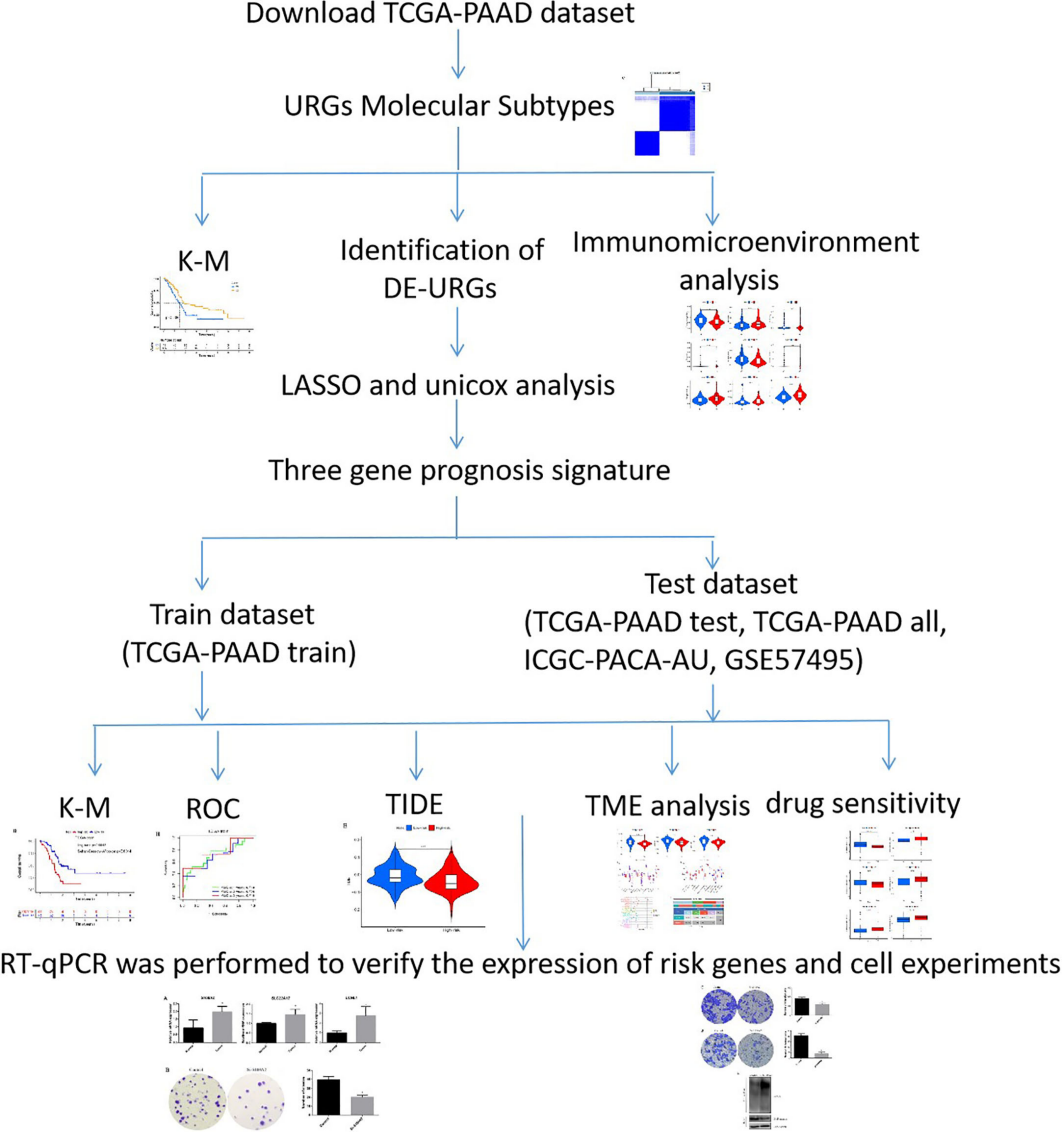
The flowchart of the current study. *P < 0.05.

The TCGA database (TCGA-PAAD, https://portal.gdc.cancer.gov, 2022.11.21) was searched to obtain the gene expression data (FPKM) of 178 PDAC tumor samples, 4 normal tissue samples, and the related clinical data. Genotype-Tissue Expression (GTEx) database (https://commonfund.nih.gov/GTEx) was used to obtain the gene expression data of pancreatic normal tissue. TCGA-PAAD was categorized into the train and test groups according to the 1:1 ratio with R software (**Supplementary Table S1**). The ICGC database (ICGC-PACA-AU, https://dcc.icgc.org/) was searched, and 88 pancreatic cancer samples along with their associated prognostic data were retrieved for external verification after normalization. The GEO database (GSE57495, https://www.ncbi.nlm.nih.gov/geo, 2022.11.21) was also obtained for external verification. A search of the MSigDB database (http://www.broad.mit.edu/gsea/msigdb/, 2022.11.21) yielded 79 URGs, and the genes are listed in **Supplementary Table S2**. The research flowchart was shown in **Figure 1**.

### Consensus clustering analysis of URGs

The R package “limma” and “ConsensusClusterPlus” were used for consistent cluster classification of PDAC (14). The association between clusters and overall survival (OS) was analyzed by R packet “survival”. The results were analyzed by R packages “pheatmap”, “survival” and “survminer” as heat maps and Kaplan-Meier (KM) curves. The “limma” program was employed to determine DEGs between two clusters with the criteria of |log fold change (FC)| >1 and FDR < 0.05. Scores of infiltrating immune cells were derived via the CIBERSORT method, and the difference in infiltration of immune cells between the two subtypes was assessed by “limma” package.

### Development and verification of the URGs prognostic signature

A univariate Cox regression analysis was conducted to determine DEGs associated with prognosis. Then, using multivariate Cox and least absolute shrinkage and selection operator (LASSO) regression analyses by “caret”, “glmnet” and “survival” packages, thress genes were selected and integrated into the prognostic signature (15). The median risk score was used to classify individuals with PDAC into two categories (low- and high-risk categories). Subsequently, the OS was compared by KM analysis. OS and Receiver Operating Characteristics (ROC) of subgroups were analyzed with the “survival”, “survminer” and “timeROC” R packages for 1, 3, and 5 years. Specifically, the “ggplot2” R program was employed to conduct a principal component analysis (PCA). By incorporating risk assessment with clinical data, a nomogram was developed. Next, multifactor ROC was implemented to verify the predictive accuracy of the nomogram.

### Comparative analysis of the tumor microenvironment between high- and low-risk categories

Immune cell abundance (ImmuneScores) and stromal cell abundance (StromalScores) were evaluated by the ESTIMATE (16). To examine the variation in immune cell infiltration between high-risk and low-risk categories, we used the TIMER, CIBERSORT-ABS, QUANTISEQ, EPIC, MCPCOUNTER, and CIBERSORT, XCELL, algorithms. The correlation of risk score and immune cell was evaluated by “corrplot” packages. Differential immune cell infiltration and immune function were probed via single-sample gene set enrichment analysis (ssGSEA) using “GSEABase” package. The expression patterns of immune-related genes were also determined. Tumor immune dysfunction and exclusion (TIDE) acted as a vital biomarker for immunotherapy response. Additionally, we assessed whether there was any link between TIDE scores and risk scores.

### Pathway analysis of the URGs signature

We examined the DEGs in the high- and low-risk categories. The underlying pathway analysis associated with DEGs was enriched through the Gene Ontology (GO), Disease Ontology (DO), and Kyoto Encyclopedia of Genes and Genomes (KEGG) analysis using “DOSE”, “org.Hs.eg.db” R packages. To assess the probable biological functioning differences between high- and low-risk categories, a gene set variation analysis (GSVA) was carried out.

### Drug sensitivity analysis

We investigated the potential for URGs signature to serve as a predictor for medications used in chemotherapy and targeted treatment. Subsequently, the half-maximal inhibitory concentration (IC50) was computed with the “pRRophetic” method (1, 8).

### Reverse transcription quantitative polymerase chain reaction

Pancreatic tissue samples were collected from the Ningbo First Hospital, including eight normal pancreatic tissue samples and eight pancreatic cancer tissue samples. The study was approved by the Ethics Committee of the Ningbo First Hospital. All research was performed in accordance with relevant guidelines/regulations. Trizol was employed to isolate total RNA, after which it was reverse-transcribed into the cDNA template. Next, RT-qPCR was conducted with the aid of SYBR Green Real-Time PCR Master Mix Plus (Toyobo). Analyses were conducted according to MIQE guidelines. The internal reference gene utilized was β-Actin. **Supplementary Table S3** outlines the amplification primer sequences.

### Cell culture and S100A2-knockdown by siRNA

PANC-1 cells were cultured in DMEM with 10% fetal bovine serum under standard culture conditions. Using Lipofectamine 2000 (Invitrogen), siRNA (100 nm) was transfected into cells 48 hours after transfection according to manufacturer’s instructions. Colony formation assay was evaluated by crystal violet staining methods. PANC-1 cells were seeded at 1000 cells per well to six‐well plates then cultivated for 14 days. Sequentially, cell number at each well was counted after staining.

### Transwell assay

Transfected PANC-1 cells were seeded at 2× 10^5^ cells per upper transwell chamber, cultivated with or without 100 mL of reconstituted Matrigel-coated membrane for 36-48 hours. Then stained the cells with crystal violet. Thereafter, number of migration or invasion cells was count.

### Statistical analysis

Data were presented as the mean ± SEM. Significant differences were evaluated by performing Student’s t-test using Prism software v6.02. Moreover, the Kruskal-Wallis test was used for variables with more than two groups. The Kruskal-Wallis test and Wilcoxon rank sum test were applied to analyze correlations. Correlation analysis between two groups of variables was used spearman correlation coefficient. Statistical significance was set at P < 0.05.

## Results

### Identification of URGs clusters in PDAC

**Figure 2:**
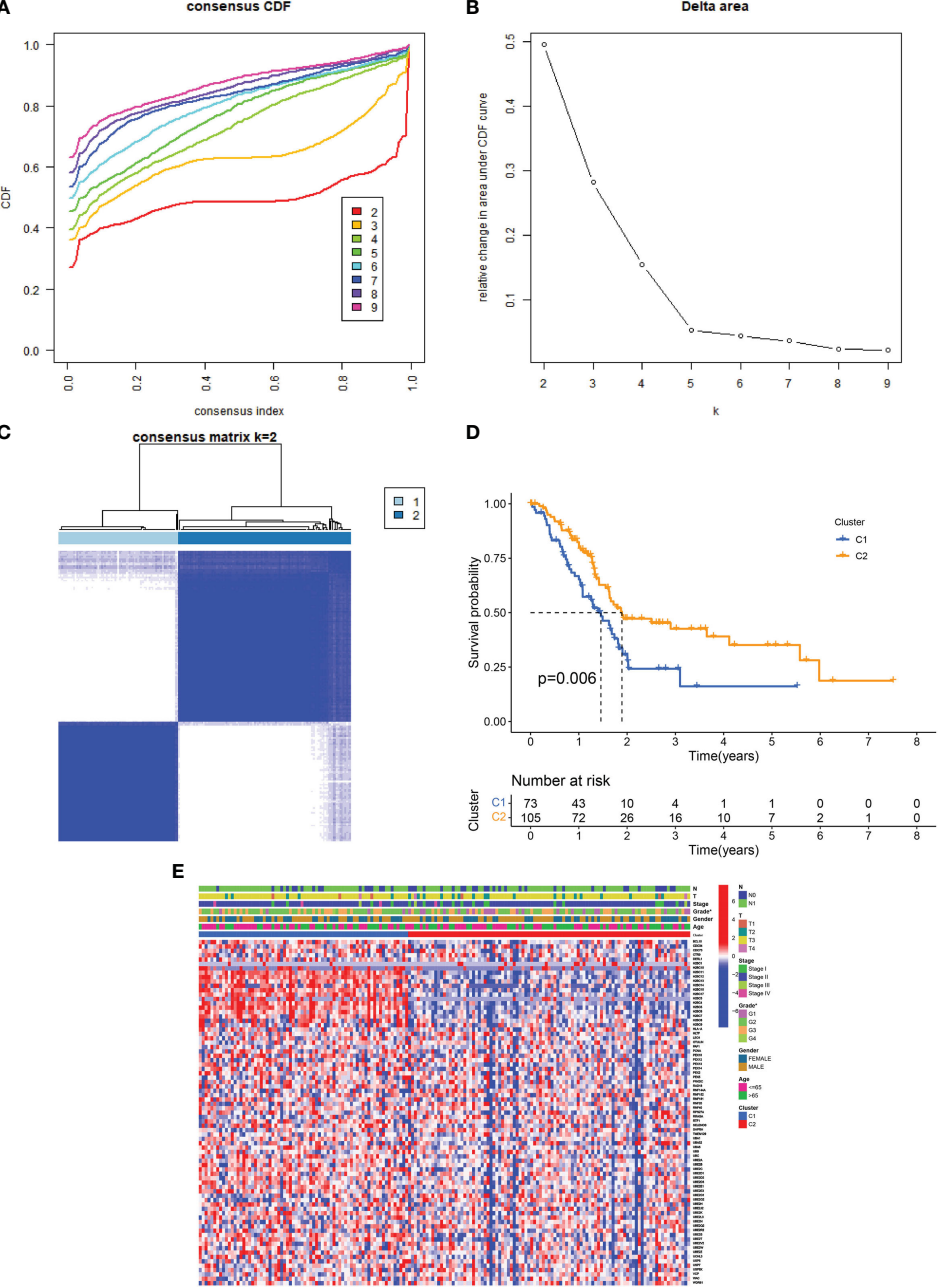
URG clusters and clinical characteristics between PDAC samples in two clusters. **(A, B)** The cumulative distribution function curve illustrates the most effective way of URG clustering. **(C)** The consensus matrix of the clustering analysis via k-means clustering (k = 2). **(D)** Kaplan–Meier (KM) curves for the overall survival (OS) of PDAC patients among different URG groups. **(E)** Heatmap of URG expression in PDAC patients with different clinical characteristics and URG clusters.

The link between URGs expression and PDAC subtypes was first analyzed using a consensus clustering method. As depicted in **Figures 2A–C**, the CDF curve was applied to categorize patients with PDAC into two clusters (C1 and C2). In contrast with C2, C1 individuals diagnosed with PDAC had remarkably lower survival duration (**Figure 2D**). The correlation between URG clusters, clinical characteristics, and URGs expression in PDAC patients is depicted in **Figure 2E**. Most URGs were expressed higher in C1 than in C2, and the Grade of the samples in C1 was higher, while T, N, stage, age and gender had no significant difference between C1 and C2.

Since immune cells perform an instrumental function in the onset and advancement of PDAC, we next evaluated the variations in infiltrating immune cells between the two clusters. In cluster 2, the level of monocytes, resting mast cells, naive B cells, and CD8 T cells were higher than in cluster 1, while Tregs, Eosinophils, Macrophages M0 and Mast cells activated were lower than in cluster 1 (**Supplementary Figure S1**).

### Development and validation of the ubiquitination-related prognostic signature

**Figure 3:**
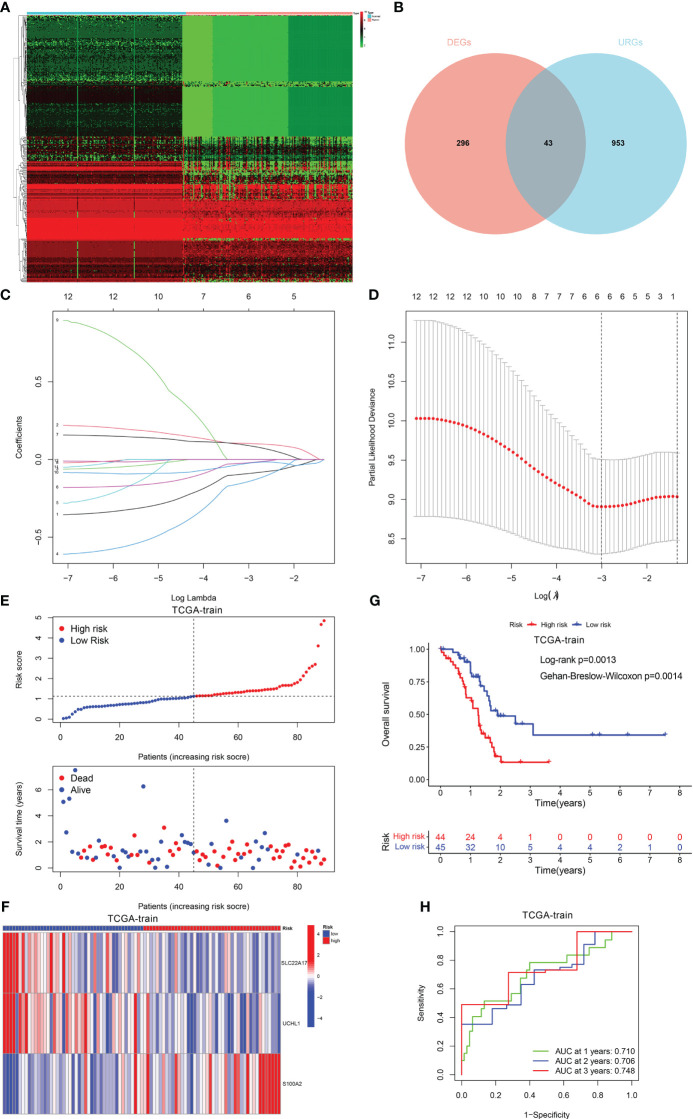
Construction of the prognostic signature. **(A, B)** The different expression of DEGs between PDAC and normal tissue. **(C)** LASSO coefficient profiles (y-axis) of the gene sets and the optimal penalization coefficient (l) via 3-fold cross-validation based on partial likelihood deviance. **(D)** The dotted vertical lines represent the optimal values of l. The top x-axis has the numbers of gene sets, whereas the lower x-axis revealed the log (*λ*). **(E)** Risk score and survival outcome of each case. **(F)** Heatmap showed the expression of 2 genes in two risk groups. **(G)** The KM curve showed that patients in the high-risk group had a worse prognosis. **(H)** The AUC for 1-, 2- and 3-years survival.

Using the “limma” program, 996 DEGs were found between two clusters with the criteria of |log fold change (FC)| >1 and FDR < 0.05. Thereafter, 43 ubiquitination-related DEGs whose expression levels were remarkably different between PDAC and normal tissues were identified (**Figures 3A, B**). Next, 12 prognosis-related DEGs were found by the univariate Cox analysis. Subsequently, we completed a LASSO analysis to remove the overfitting genes and the URGs signature of 3 genes (SLC22A17, UCHL1 and S100A2) was created (**Figures 3C, D**). The equation applied to derive the risk score is as indicated: risk score= (SLC22A17 × (-0.260926538020362) + (UCHL1 × (-0.286371148071792) + (S100A2 × (0.157355046660652).

Patients with PDAC were classified into low- and high-risk categories as per the median risk score value (**Figure 3E**). The variations in the expression of these two genes between the two risk categories are illustrated in **Figure 3F**. Also, patients having elevated risk scores had a greater fatality rate (**Figure 3G**). Moreover, the ROC curve was performed to assess the URGs signature, which manifested that the AUC values for 1-, 2- and 3-year periods were 0.710, 0.706, and 0.748, respectively (**Figure 3H**).

**Figure 4:**
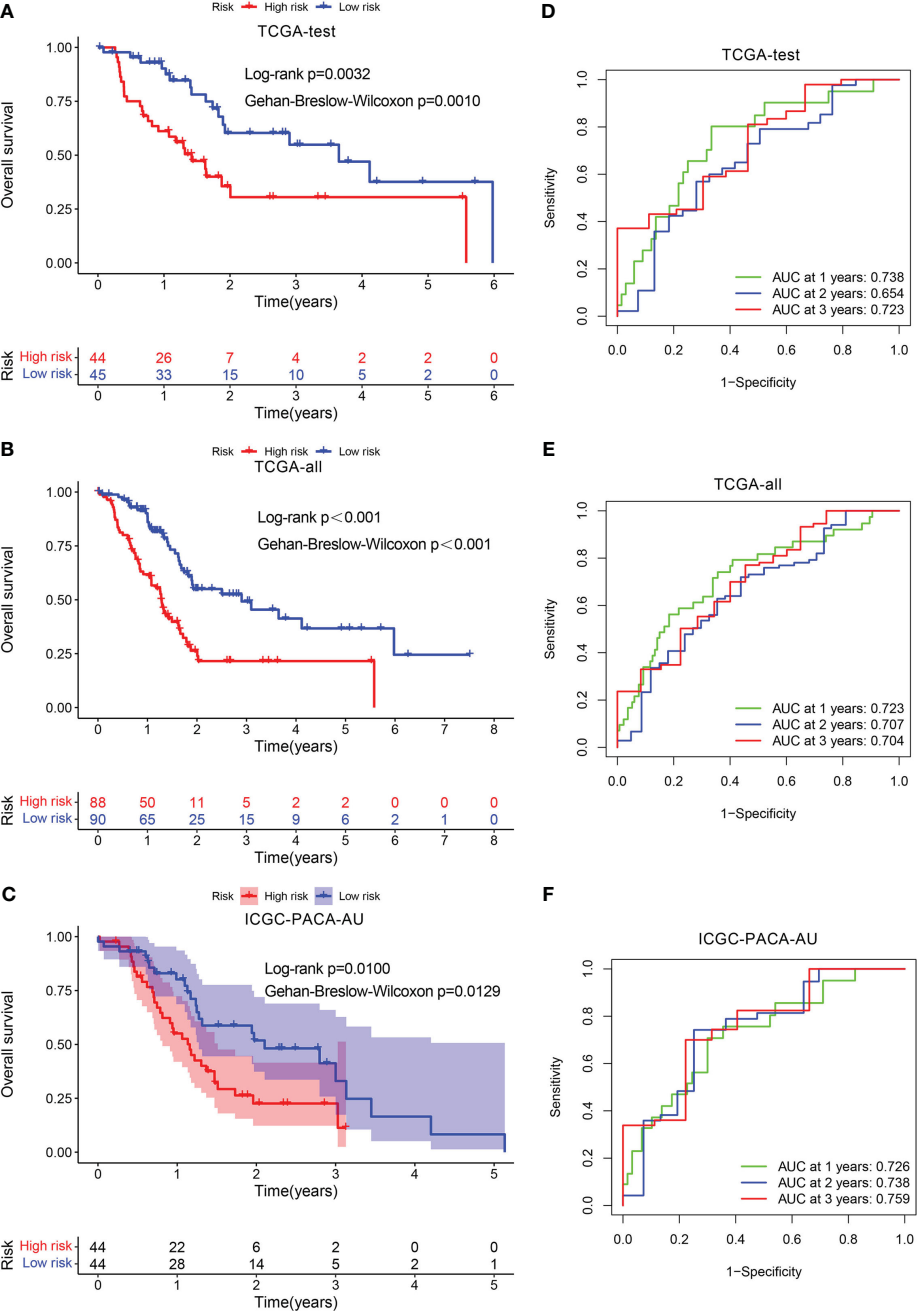
Validation of the prognostic signature. KM curve showed that patients in the high-risk group had a worse prognosis in TCGA-test **(A)**, TCGA-all **(B)**, and ICGC-PACA-AU **(C)**. The AUC for 1-, 2- and 3-years survival in TCGA-test **(D)**, TCGA-all **(E)**, and ICGC-PACA-AU **(F)**.

Furthermore, we verified the aforementioned findings in test datasets. All patients with PADC in the test datasets were also classified into low- and high-risk categories. The KM analysis disclosed that the low-risk individuals exhibited a more favorable prognosis in contrast to those at high risk in TCGA-test, TCGA-all, and ICGC-PACA-AU (**Figures 4A–C**). The AUC values of the ROC curve of 1-, 2-, and 3-year periods were 0.738, 0.654, and 0.723, correspondingly, in TCGA-test (**Figure 4D**), 0.723, 0.707, and 0.704 in TCGA-all (**Figure 4E**), and 0.726, 0.738, and 0.759 in ICGC-PACA-AU (**Figure 4F**). In addition, another test dataset GSE57495 was also used for validation. Patients having high risk scores had a greater fatality rate, the AUC values for 1-, 2- and 3-year periods were 0.706, 0.733, and 0.871, respectively (**Supplementary Figure S2**).

### Construction of a nomogram for PDAC

**Figure 5:**
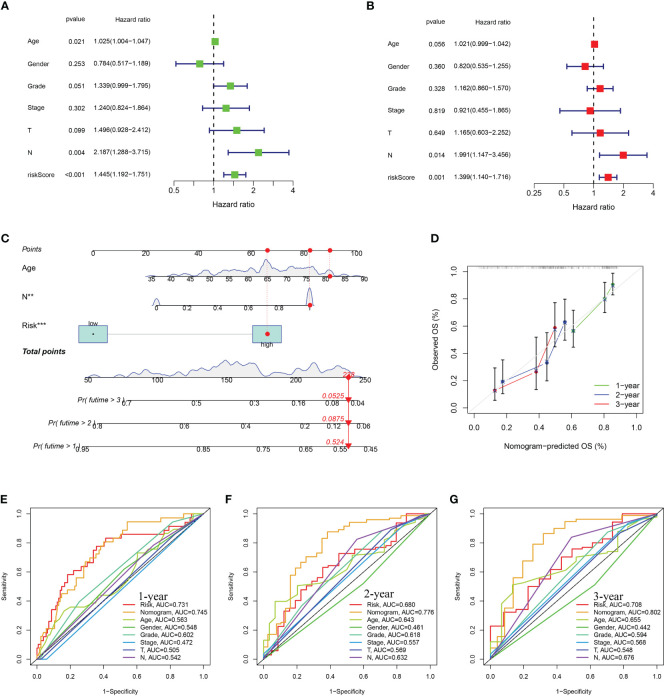
Construction and assessment of nomogram. **(A)** Univariate Cox regression **(B)** multivariate Cox regression analyses. **(C)** The prediction of nomogram in the TCGA-train dataset. **(D)** Calibration plots for the nomogram. The multifactor AUC for 1- **(E)**, 2- **(F)**, and 3-years **(G)** survival.

Multivariate and univariate Cox regression analyses proved that risk score independently acted as a robust prognostic marker (P < 0.05) (**Figures 5A, B**). An innovative nomogram was developed using the URG signature and clinical variables from the training dataset to further exploit the URG signature’s prognostic potential (**Figure 5C**). The predictive 1, 2, 3-survival rate was close to the actual observation (**Figure 5D**). In addition, the ROC analysis was conducted to evaluate the nomogram’s prognosis-predicting value. For 1-year survival times, the AUC value was 0.745 (nomogram), 0.731(risk score) (**Figure 5E**). For 2-year survival times, the AUC value was 0.776 (nomogram), 0.680(risk score) (**Figure 5F**). For 3-year survival times, the AUC value was 0.802 (nomogram), 0.708 (risk score) (**Figure 5G**). These results revealed that this novel nomogram could act as an admirable prognosis prediction model.

### The tumor microenvironment analysis in high- and low-risk groups

**Figure 6:**
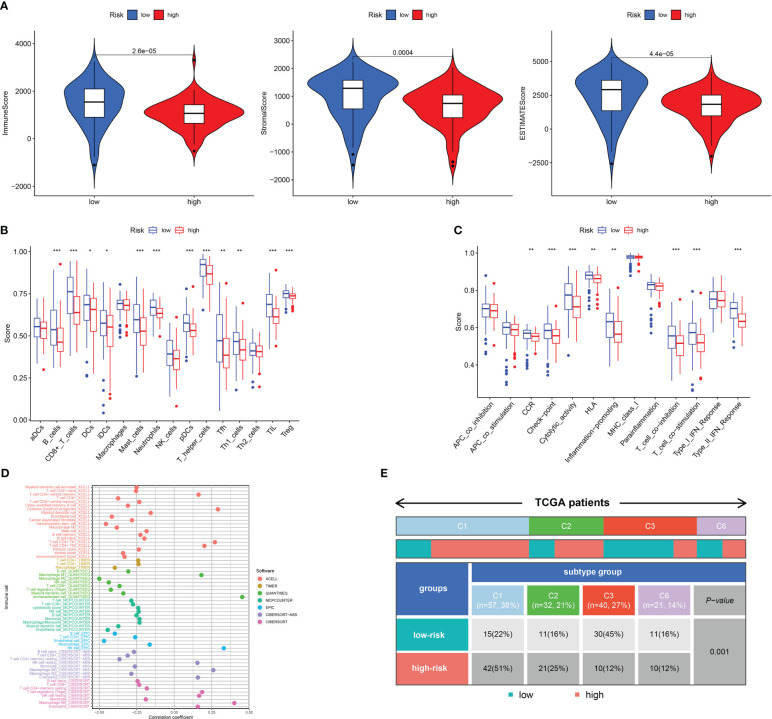
Analysis of immune conditions of high- and low-risk groups. **(A)** Differences in immune microenvironment scores between the two groups. **(B)** The analysis of differences in immune cell infiltration between the two groups with ssGSEA. **(C)** The analysis of differences in immune functions between the two groups with ssGSEA. **(D)** The analysis of differences in immune cell infiltration between the two groups with Multiple algorithms. **(E)** The distribution of patients with high- and low-risk in different immune subtypes. *P <0.05; **P <0.01; ***P <0.001.

The TME serves as a crucial indicator of the biological behavior of the tumor. ESTIMATE analysis revealed that the ImuneScores, StromalScores, and ESTIMATEScores were all lower in the high-risk category in contrast with the low-risk category (**Figure 6A**). ssGSEA analysis found less infiltration of the B cells, CD8+ T cells, DCs, iDCs, Neutrophils, Mast cells, T helper cells, Tumor-infiltrating cell (TIL), and T cells regulatory (Treg) in the high-risk patients in contrast with the low-risk patients (**Figure 6B**). Some immunologic functions, including T cells co-stimulation, CCR, Type II IFN response, and T cell co-inhibition were also improved in the low-risk patients (**Figure 6C**). Additionally, the distinctions of immune cell levels between the two risk groups were also investigated through CIBERSORT, MCPCOUNTER, QUANTISEQ, EPIC, TIMER, CIBERSORT-ABS, and XCELL. As per the findings, the low-risk category had remarkably higher levels in most immune cells, including naive CD4 T cells, CD8 T cells, DCs, Cancer associated fibroblast, NK cell, B cell and Monocyte (**Figure 6D**). This may explain why the low-risk category has a superior prognosis. Additionally, **Figure 6E** depicted the distribution of low- and high-risk individuals across multiple immune subtypes.

**Figure 7:**
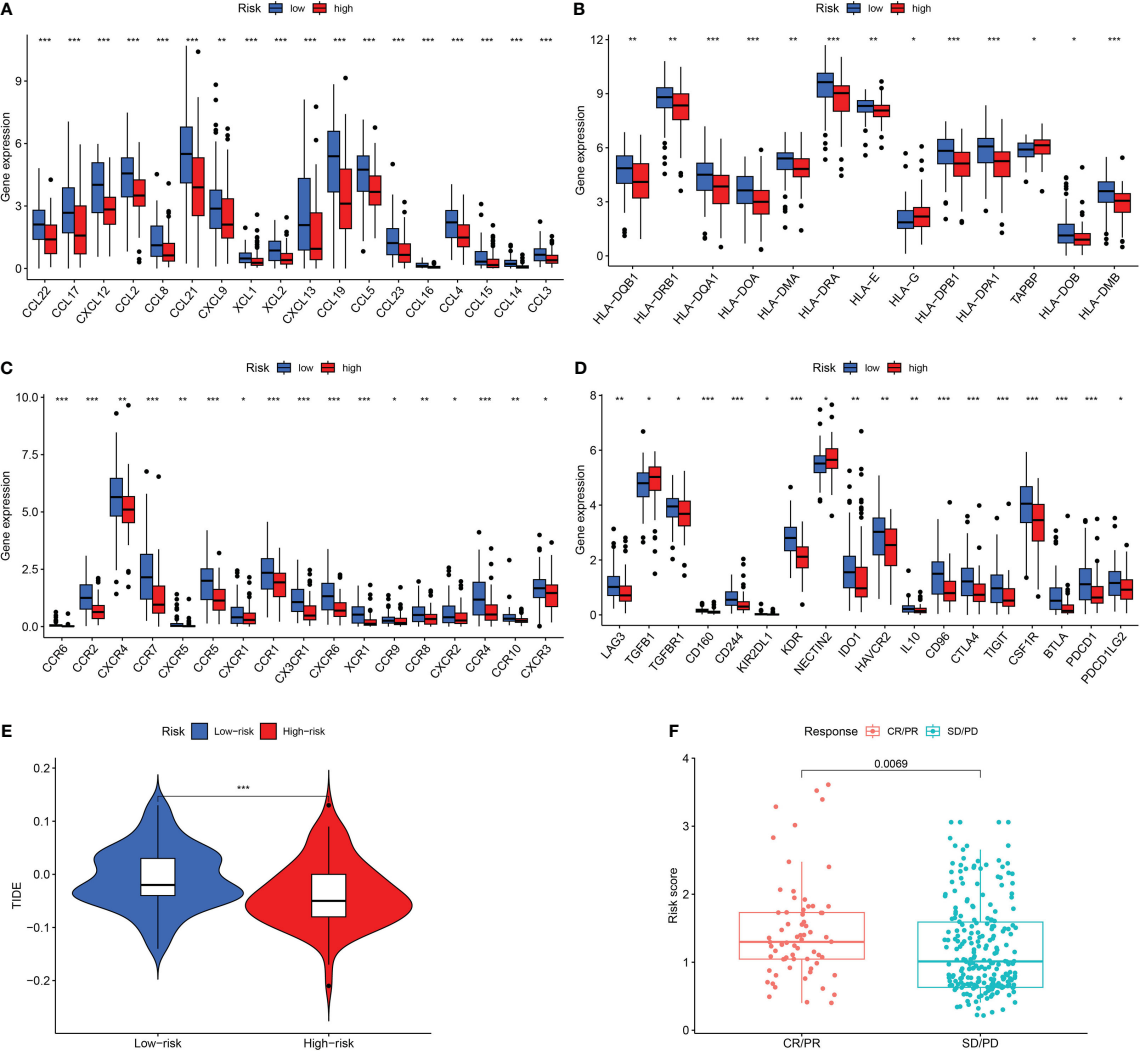
Assessment of Immunotherapy response of high- and low-risk groups. **(A–D)** The immune-related gene expression levels in different groups. **(E)** The TIDE scores in different groups. **(F)** Prediction immunotherapy response in IMVigor210. *P <0.05; **P <0.01; ***P <0.001.

We next examined the low- and high-risk patients in terms of the expression patterns of immune-related genes. A majority of immune-related genes were discovered to be expressed at low levels in the high-risk category (**Figures 7A—D**). TIDE scores acted as a vital biomarker for immunotherapy response. The link between the TIDE score and risk score was also investigated. TIDE scores were found to be lower in the high-risk category in contrast with the low-risk category (**Figure 7E**). Furthermore, high-risk patients respond better to immunotherapy in contrast with those at low risk (**Figure 7F**).

### Comparative analysis of mutations and functional evaluation of the URG signature

**Figure 8:**
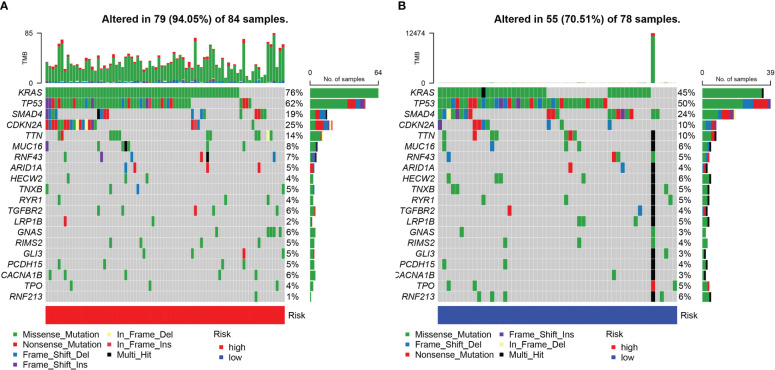
The somatic gene mutations in the high-risk group **(A)** and low-risk group **(B)**.

**Figure 9:**
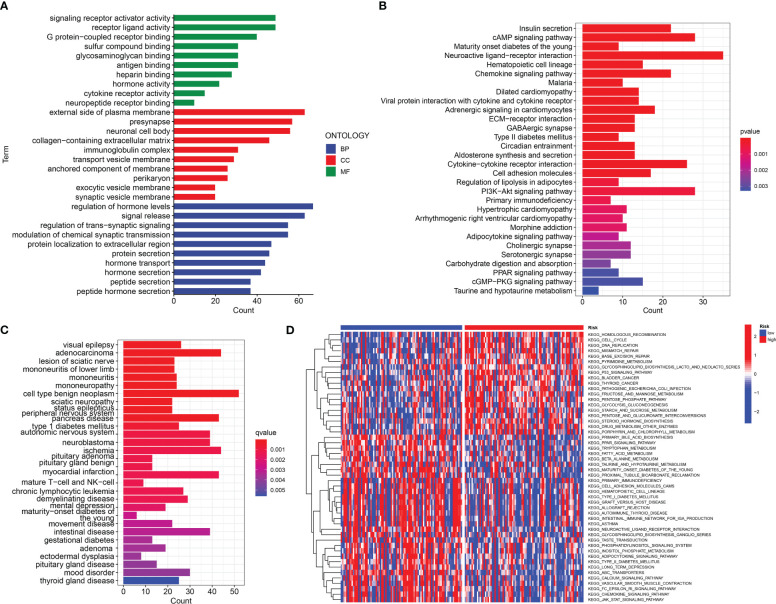
Function analysis. **(A)** GO analysis of differential genes between high and low-risk groups. **(B)** KEGG analysis of differential genes between high- and low-risk groups. **(C)** DO analysis of differential expression genes. **(D)** GSVA enrichment analysis in high- and low-risk groups.

The somatic mutations of PDAC with high and low URG scores were examined to adequately describe the pathobiological significance of the URG score. The high-URG score group had a greater somatic mutation frequency (94.05%) in contrast with the low-URG score group (70.51%) (**Figures 8A, B**). The GO, KEGG, and DO enrichment analyses were conducted to investigate the latent biological roles of the DEGs. Accordingly, the GO result revealed that the DEGs were primarily enriched in signal release, external side of plasma membrane, and antigen binding (**Figures 9A, C**). The KEGG result suggested that the DEGs were primarily enriched in insulin secretion, cAMP signaling pathway, and Chemokine signaling pathway (**Figure 9B**). The DO result suggested that the DEGs were primarily enriched in cell type benign neoplasm, adenocarcinoma and pancreas disease (**Figure 9C**). In addition, the GSVA indicated that many pathways were substantially altered between the high- and low-risk PDAC patients (**Figure 9D**).

### The correlation analysis of drug sensitivity and risk score

**Figure 10:**
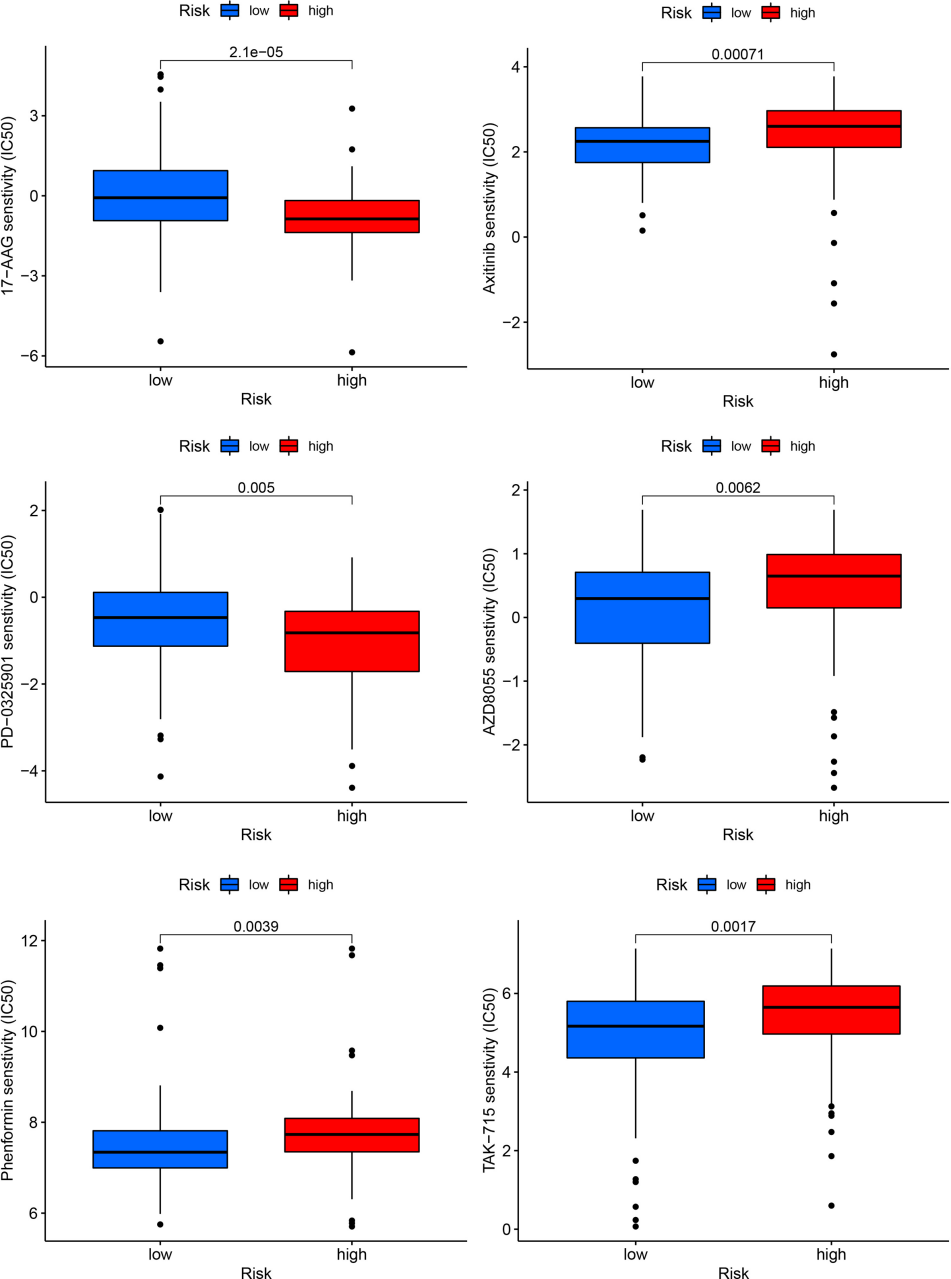
Drug sensitivity analysis in high and low-risk groups.

We correlated the PDAC patients’ risk scores with the IC50 values of chemotherapy and targeted treatment medications to learn more about the possible variations in drug sensitivity between low- and high-risk categories. The IC50 values of 17-AAG and PD-0325901 were significantly higher in low-risk group, whereas the IC50 values of Phenformin, Axitinib, AZD8055 and TAK-715 were lower in low-risk group (**Figure 10**).

### Knockdown of S100A2 inhibits the malignant biological behavior of pancreatic cancer

**Figure 11:**
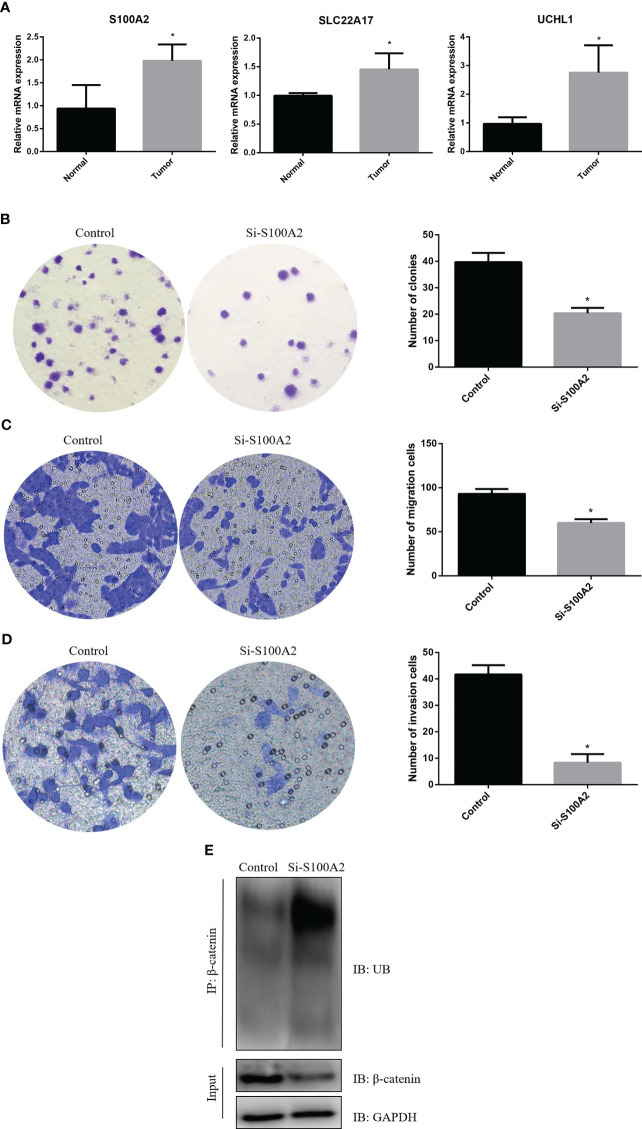
S100A2 knockdown inhibits the malignant biological behavior of PDAC cell. **(A)** Detection of risk gene expression in pancreatic cancer tissues and normal tissues by RT-qPCR. **(B)** S100A2 knockdown inhibits the cloning of PANC-1 cell. **(C)** S100A2 knockdown inhibits the migration ability of PANC-1 cell. **(D)** S100A2 knockdown inhibits the invasion ability of PANC-1 cell. **(E)** S100A2 knockdown increased the ubiquitination of β-catenin. *P <0.05.

First, we detected the expression of three risk genes by RT-qPCR. The results showed that the expressions of three risk genes in pancreatic cancer tissue were higher than normal pancreatic tissue (**Figure 11A**). Next, we knocked down of S100A2 in PANC-1 cells to explore the role of S100A2 in pancreatic cancer. The results showed that S100A2 knockdown reduced the cloning, migration, and invasion ability of PANC-1 cells (**Figures 11B–D**). In addition, it was found that S100A2 knockdown increased the ubiquitination of β-catenin, thereby reducing its protein expression (**Figure 11E**). These results suggested that S100A2 knockdown might inhibit the malignant biological behavior of pancreatic cancer cells by increasing the ubiquitination of β-catenin.

## Discussion

Pancreatic cancer is a particularly deadly malignancy of the digestive system (18). Although there is a new understanding of the molecular mechanism of PDAC, and new progress in surgery, adjuvant therapy, and chemotherapy, patients diagnosed with PDAC have not seen a substantial improvement in their prognoses (19). For a long time, the prognosis of PDAC has been judged mainly based on clinical manifestations, tumor stage, pathological grade, lymph node metastasis, neurovascular invasion, and other pathological characteristics (19, 20). Consequently, predicting a patient’s prognosis in PDAC using these markers is challenging. Additionally, in the early diagnosis of PDAC, the detection of single tumor markers, such as CEA, CA199, and CA125, is difficult to reach the ideal level of sensitivity and specificity (21–26), which can no longer meet the current clinical needs. Recently, Wu et al. found a nine-gene signature, which could anticipate the OS duration of PDAC patients (27). As per the expression of immune-related genes such as MMP14 and INHBA, Xu et al. developed a predictive risk model to assess PADC patients’ prognoses and identify PDAC therapy opportunities (28). However, there is much less understanding of ubiquitination in the PDAC prognosis and microenvironment. Here, we built a risk model using three SLC22A17, UCHL1 and S100A2, to indicate the outcomes of PDAC. The risk model also presented as a potential biomarker to reflect the sensitivity of targeted therapy and immune status in tumor tissues. Our study analyzed the mRNA expression data of these three risk model genes.

First, we identified 996 DEGs between the two clusters of ubiquitinated subtypes, and further screened 12 genes correlated with the prognosis from these DEGs utilizing univariate Cox regression analysis. Thereafter, we utilized LASSO regression analysis to obtain the optimal genes for predicting outcomes. Lastly, a prognostic risk model of PDAC containing three ubiquitination-related genes (SLC22A17, UCHL1 and S100A2) was constructed, and KM and ROC curve analyses were employed in TCGA, ICGC and GEO data sets to confirm the effectiveness of the model for prognostic evaluation of PDAC. Wei et al. found that high expression of SLC22A17 indicates poorer prognosis in gastric cancer (29). UCHL1 might play a role in the malignant progression of triple-negative breast cancer by maintaining dryness and promoting cell invasion (30). Li et al. revealed S100A2 promoted glycolysis and proliferation of colorectal cancer through GLUT1 regulation (31). In this study, we found S100A2 knockdown could inhibit the malignant biological behavior of pancreatic cancer cells by increasing the ubiquitination of β-catenin. However, the significance of other two genes in PDAC remains uncertain and this is a direction of pancreatic cancer research in the future.

The tumor microenvironment (TME) is constituted of diverse immune cells, interstitial cells, extracellular matrix, and tumor blood vessels, which stimulate the onset and advancement of cancer (31). The infiltrating immune cell levels in TME usually change with tumorigenesis and progression (32). Our analysis illustrated that PDAC patients having high risk scores recorded lower ImuneScores, StromalScores and ESTIMATEScores. We found that most of the immune cells (B cells, CD8+ T cells, Treg, T helper cells, Neutrophils, TIL, and Mast cells) were substantially reduced in the high-risk patients in contrast with the low-risk patients. Additionally, the majority of immune-related genes tended to be downregulated in the high-risk population, whereas the low-risk category illustrated considerable improvement in immunologic functioning. Research suggests that immune cells are important components of anti-tumor immunity (33). One reason high-risk individuals have such a dismal prognosis is that they have fewer immune cells and attenuated immunological functioning. TIDE scores acted as vital biomarkers for immunotherapy response (34). Our results highlighted that high-risk individuals with PDAC responded more positively to immunotherapy compared to those in the low-risk category and that the TIDE scores were lower in the high-risk patients in contrast with those in the low-risk category. The findings of this research shed light on the involvement of ubiquitination in PDAC and may be utilized to direct immunotherapeutic and chemotherapeutic interventions for PDAC patients.

Nevertheless, our investigation does have a few drawbacks. Case selection bias could be present since the vast majority of analyses use data from publicly available data sets and all samples are retrieved retroactively. Furthermore, additional *in vitro* and *in vivo* tests are warranted to corroborate our findings.

In summary, we designed a molecular cluster and prognostic signature based on URGs, which aid in anticipating survival, directing immunotherapy, and determining clinical outcomes. This research potentially provides deeper insights into the function of ubiquitination in PDAC and facilitates the development of more effective therapies for this disease.

## Data availability statement

The original contributions presented in the study are included in the article/**Supplementary Material**. Further inquiries can be directed to the corresponding authors.

## Author contributions

YG designed the study. LQ, YB and ZW wrote the manuscript. KC, YD, YM and KH performed data collection and curation. ZW performed RT-qPCR experiments. All authors have read and agreed to the published version of the manuscript.

## Supplementary material

Supplementary Figure 1 The differences in immune cell infiltration between two clusters.

Supplementary Figure 2 Validation of the Ubiquitination-related prognostic signature in GSE57495.
