# Supplementary figures and images for: Establishment and validation of a ubiquitination-related gene signature associated with prognosis in pancreatic duct adenocarcinoma
Source: Front Immunol. 2023 Jun 9;14:1171811. doi: 10.3389/fimmu.2023.1171811 (PMC10289160; doi:10.3389/fimmu.2023.1171811)

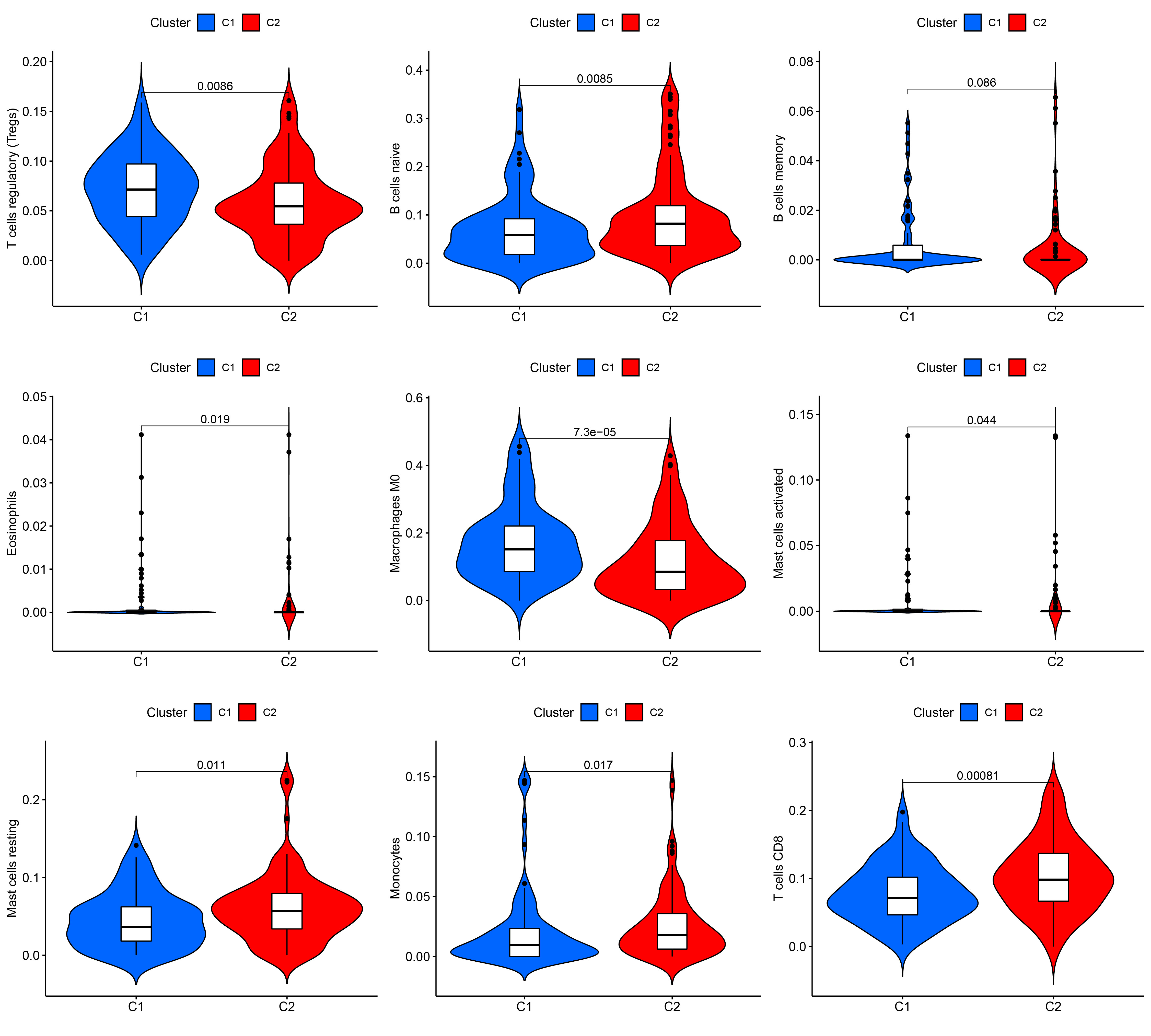

Supplement: Supplementary Figure 1 — The differences in immune cell infiltration between two clusters. [file Image_1.jpeg]

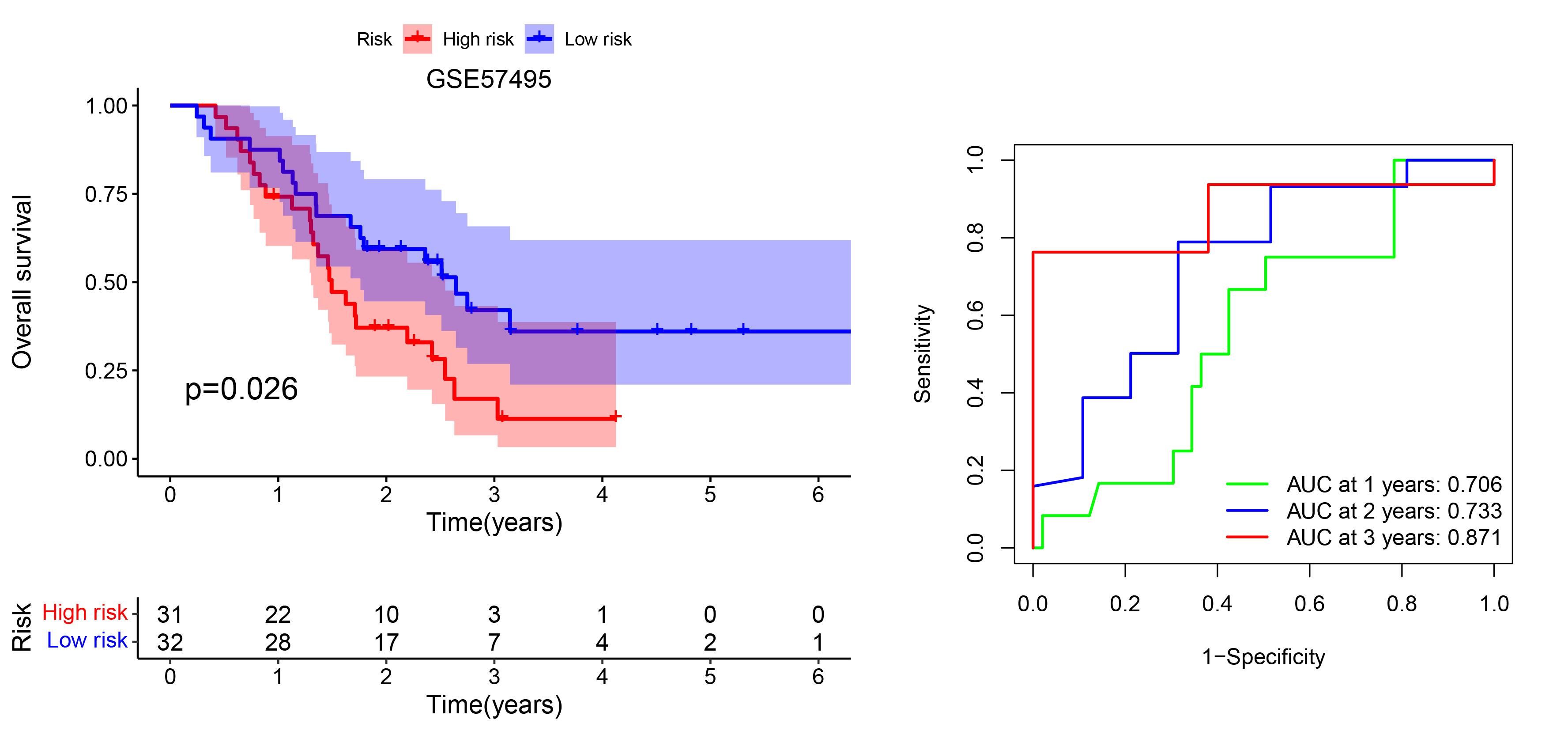

Supplement: Supplementary Figure 2 — Validation of the Ubiquitination-related prognostic signature in GSE57495. [file Image_2.jpeg]
